# Accuracy of a whole‐body single‐photon emission computed tomography with a thallium‐bromide detector: Verification via Monte Carlo simulations

**DOI:** 10.1002/mp.17724

**Published:** 2025-02-27

**Authors:** Toshimune Ito, Keitaro Hitomi, Michael Ljungberg, Sousei Kawasaki, Yuka Katayama, Akane Kato, Hirotatsu Tsuchikame, Kentaro Suzuki, Kyosuke Miyazaki, Ritsushi Mogi

**Affiliations:** ^1^ Department of Medical Radiological Faculty of Medical Technology Teikyo University Tokyo Japan; ^2^ Department of Quantum Science and Energy Engineering Graduate School of Engineering Tohoku University Sendai Japan; ^3^ Medical Radiation Physics Lund University Lund Sweden; ^4^ Department of Radiology, Nippon Medical School Hospital Tokyo Japan; ^5^ Department of Radiological Technology, Showa University Hospital Tokyo Japan; ^6^ Department of Radiology Institute of Science Tokyo Hospital Tokyo Japan; ^7^ Department of Radiology, Saiseikai Yokohamashi Tobu Hospital Yokohama Kanagawa Japan; ^8^ Department of Radiological Technology, Toranomon Hospital Tokyo Japan; ^9^ Department of Radiology, Kawasaki Municipal Kawasaki Hospital Kawasaki Kanagawa Japan

**Keywords:** development (new technology and techniques), instrumentation, Monte Carlo modeling, phantoms–digital

## Abstract

**Background:**

Single‐photon emission computed tomography (SPECT) devices equipped with cadmium–zinc–telluride (CZT) detectors achieve high contrast resolution because of their enhanced energy resolution. Recently, thallium bromide (TlBr) has gained attention as a detector material because of its high atomic number and density.

**Purpose:**

This study evaluated the clinical applicability of a SPECT system equipped with TlBr detectors using Monte Carlo simulations, focusing on 99mTc and 177Lu imaging.

**Methods:**

This study used the Simulation of Imaging Nuclear Detectors Monte Carlo program to compare the imaging characteristics between a whole‐body SPECT system equipped with TlBr (T‐SPECT) and a system equipped with CZT detectors (C‐SPECT). The simulations were performed using a three‐dimensional brain phantom and a National Electrical Manufacturers Association body phantom to evaluate 99mTc and 177Lu imaging. The simulation parameters were accurately set by comparing them with the actual measurements.

**Results:**

The T‐SPECT system demonstrated improved energy resolution and higher detection efficiency than the C‐SPECT system. In 99mTc imaging, T‐SPECT demonstrated 1.71 times higher photopeak counts and improved contrast resolution. T‐SPECT exhibited a significantly lower impact of hole tailing and higher‐energy resolution (4.50% for T‐SPECT vs. 7.34% for C‐SPECT). Furthermore, T‐SPECT showed higher peak signal‐to‐noise ratio (PSNR) and structural similarity (SSIM) values, indicating better image quality. In 177Lu imaging, T‐SPECT showed 2.76 times higher photopeak counts and improved energy resolution (3.94% for T‐SPECT vs. 5.20% for C‐SPECT). T‐SPECT demonstrated a higher contrast recovery coefficient (CRC) and contrast‐to‐noise ratio (CNR) across all acquisition times, maintaining sufficient counts even with shorter acquisition times. Moreover, T‐SPECT acquired higher low‐frequency values in power spectrum density (PSD), indicating more accurate internal image reproduction.

**Conclusions:**

T‐SPECT offers superior energy resolution and detection efficiency than C‐SPECT. Moreover, T‐SPECT can provide higher contrast resolution and sensitivity in clinical imaging with 99mTc and 177Lu. Furthermore, the Monte Carlo simulations are confirmed to be a valuable guide for the development of T‐SPECT.

## 1 INTRODUCTION

The use of single‐photon emission computed tomography (SPECT) devices equipped with cadmium–zinc–telluride (CZT) detectors (C‐SPECT), which provide high contrast resolution because of their enhanced energy resolution, has been extensively reported. Numerous studies have highlighted its effectiveness for cardiac applications,^1^, ^2^, ^3^, ^4^, ^5^, ^6^, ^7^, ^8^ and its feasibility for whole‐body imaging has also been documented.^9^, ^10^, ^11^, ^12^ Despite demonstrating excellent charge collection efficiency at room temperature, range from 5 to 35°C and presenting enhanced energy resolution with promising prospects for Compton imaging, C‐SPECT has not yet been implemented for clinical use as Compton imaging.^13^

Recently, thallium bromide (TlBr) has gained attention as a detector material with high detection efficiency.^14^, ^15^, ^16^, ^17^ TlBr possesses a high atomic number for its constituent atoms and has a high density (7.56 g/cm^3^). At 140‐keV gamma rays, TlBr's linear attenuation coefficient is 3.3 times that of CZT and 5.1 times that of NaI, making it a semiconductor material with exceptionally high gamma‐ray absorption efficiency.

Furthermore, the carrier mobility‐lifetime product of TlBr crystals has been reported to be roughly equivalent to that of CdTe crystals.^18^ However, because TlBr crystals are ionic conductors, when a high electric field is applied at room temperature, Tl^+^ and Br^−^ ions accumulate at the cathode and anode, respectively. This can reduce the effective internal electric field of the detector, decreasing the charge acquisition efficiency due to the polarization, raising concerns about device degradation. As a countermeasure, the use of thallium metal as an electrode material and neutralizing ion accumulation beneath the electrode through an electrochemical reaction have been reported to have the ability to suppress such degradation, allowing stable operation at room temperature.^19^ Hence, clinical imaging with high contrast resolution and sensitivity is anticipated with the development of clinical SPECT devices equipped with TlBr elements using thallium as the electrode material (T‐SPECT).

In contrast, Monte Carlo simulation techniques have become useful tools for analyzing, developing, and evaluating the physical characteristics of nuclear medicine imaging devices and methods and for educational purposes.^20^, ^21^, ^22^, ^23^, ^24^ One such Monte Carlo simulation code, Simulation Medical Imaging Nuclear Detectors (SIMIND), is specialized for gamma cameras and SPECT. SIMIND is frequently used to design imaging systems that include collimators and evaluate the accuracy of reconstruction algorithms and various correction methods, such as scattering and attenuation. Moreover, it can simulate arbitrary voxelized phantom acquisition data from x‐ray computed tomography (CT) data.^25^ In this study, we first verified the Monte Carlo simulation's accuracy by comparing it with actual measurements using a 57Co source in a 2 × 2 pixel format.

Furthermore, by employing Monte Carlo simulations with accurately assured parameters for a whole‐body SPECT system, we simulated a three‐dimensional brain phantom assuming clinical cerebral blood flow SPECT using 99mTc preparations and a National Electrical Manufacturers Association (NEMA)‐body phantom simulation assuming SPECT imaging for predicting the therapeutic effect of peptide receptor radionuclide therapy for neuroendocrine tumors using 177Lu‐DOTATATE preparations. Clinical applicability was evaluated by comparing it with simulation data from a C‐SPECT, including differences in radionuclide formulations.

## 2 METHODS

### 2.1 Acquisition of data‐processing device

**FIGURE 1:**
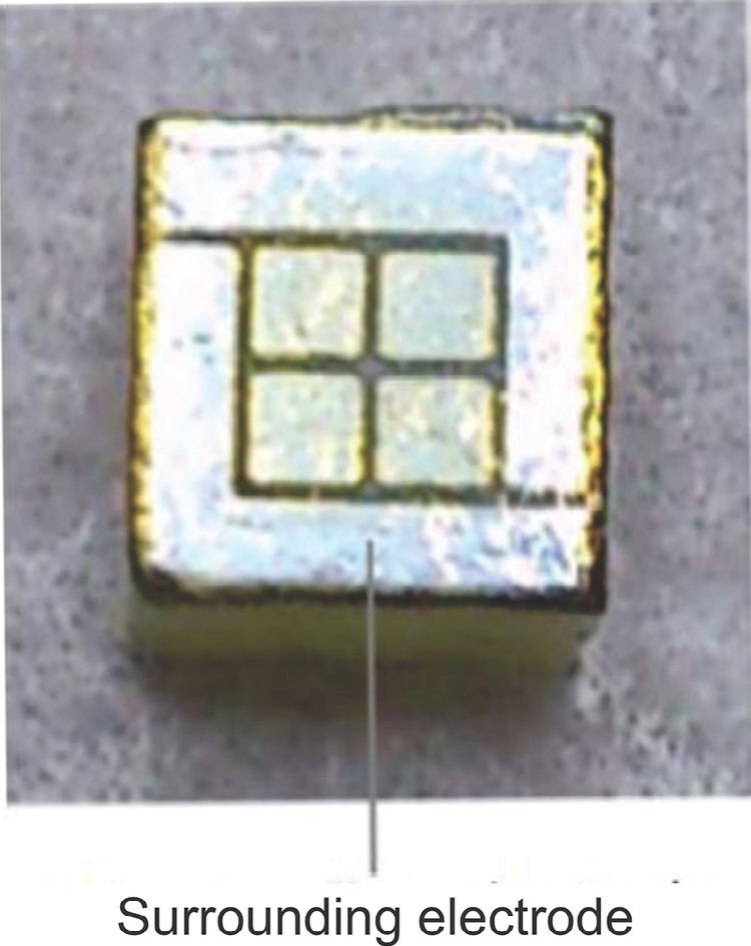
2 × 2 pixel detector with a pixel gap of 0.2 mm on a 1 × 1 mm^2^ area.^17^

The energy spectrum of a 57Co point source (1 × 1 mm^2^) in TlBr was obtained using a 2 × 2 pixel TlBr detector (1 × 1 mm^2^) fabricated by Hitomi et al. (Figure 1).^17^

A 57Co point source (1 × 1 mm^2^) and a 2 × 2 pixel TlBr detector with a detector size of 1 × 1 mm^2^ were simulated to obtain the energy spectrum. The Monte Carlo simulation code used was the SIMIND Monte Carlo program (version 7). Data from a whole‐body SPECT‐CT system equipped with a CZT detector, NM/CT 870 CZT (GE Healthcare, WI, USA), were simulated for the assumed experimental imaging. In the simulation, the detector elements were selected as CZT and TlBr. For the TlBr configuration, the physical dimensions matched those of the CZT module, except for detector thickness. The CT device used to create the digital phantom data used in the simulation was the Aquilion Prime (Canon Medical Systems, Tokyo, Japan).

**FIGURE 2:**
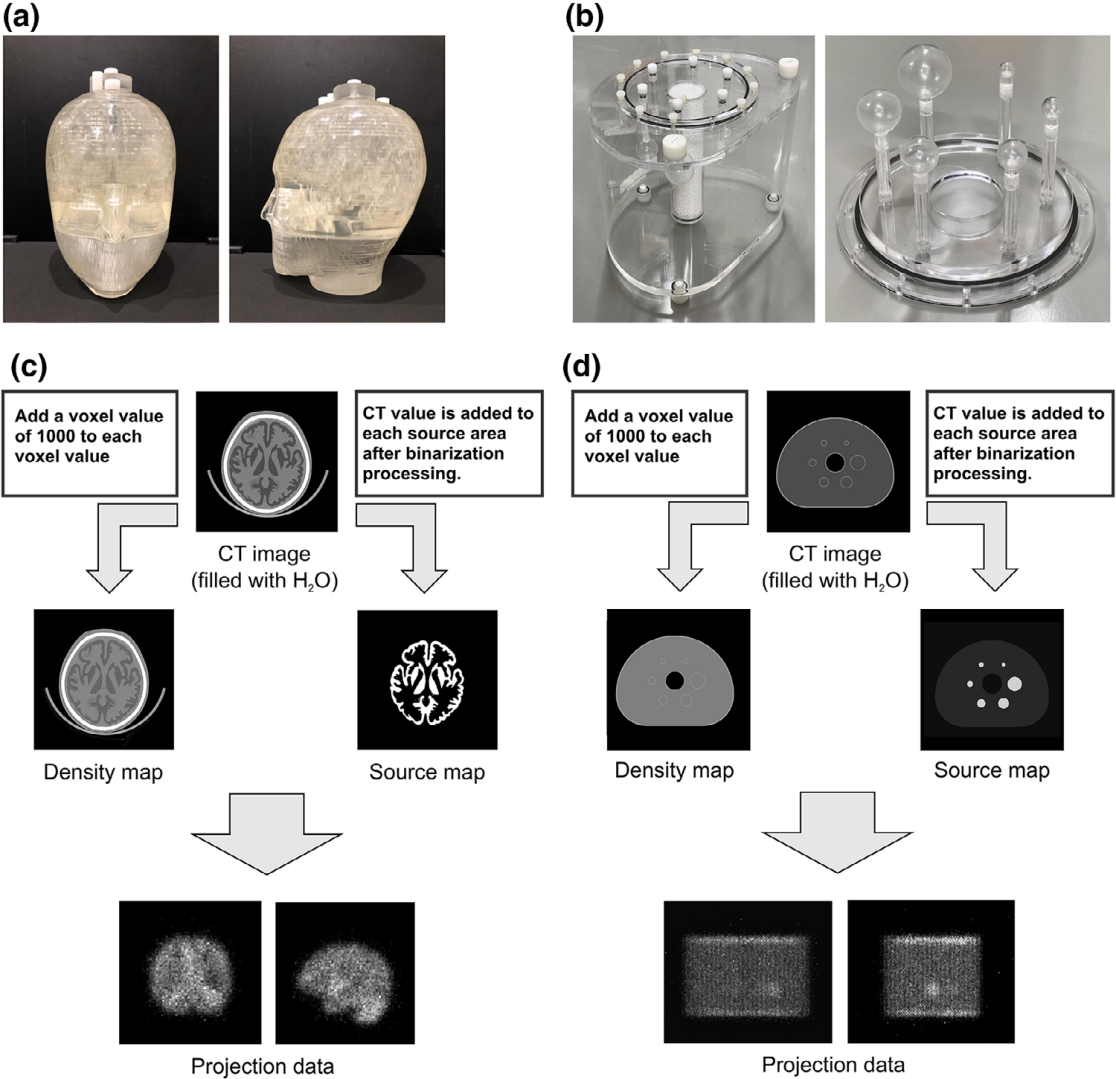
Each actual measurement phantom assumed for constructing the digital phantom. (a) Three‐dimensional brain phantom. (b) NEMA‐IEC body phantom. The phantom was modeled on a NEMA‐IEC body phantom. Spheres were installed in the phantom with diameters of 10, 13, 17, 22, 28, and 37 mm. (c) Simulation flow diagram: SPECT projection data acquisition for 3D brain phantom. (d) Simulation flow diagram: SPECT projection data acquisition for NEMA‐IEC body phantom. NEMA, National Electrical Manufacturers Association; SPECT, single‐photon emission computed tomography.

Two types of digital phantoms were created: a three‐dimensional brain phantom (Molecular Imaging Labo, Osaka, Japan) and (NEMA‐IEC) body phantom (Kyoto Chemical Industry, Kyoto, Japan) (Figure 2).

For image processing and analysis, we used Prominence Processor (version 3.1; Nihon Medi‐Physics Co. Ltd., Tokyo, Japan), ImageJ (National Institutes of Health, USA), Demon Research Image Processor (version 3.01; PDR Adiopharma Inc., Tokyo, Japan), and Xeleris (GE Healthcare, WI, USA). For statistical processing, we used EZR (Saitama Medical Center, Jichi Medical University, Saitama, Japan), which is a graphical user interface for R (The R Foundation for Statistical Computing, Vienna, Austria).^26^

### 2.2 Comparison between real acquisition and simulation

#### 2.2.1 Small‐scale pixel detector

Under ambient room temperature conditions, a 57Co source was irradiated onto a 4.36‐mm‐thick 4‐pixel TlBr pixel detector, and a gamma‐ray spectrum with depth correction was extracted from pixel #2. The cathode was biased at 500 V, and acquisition time was 120 min. Furthermore, a digital phantom under similar conditions was established, and simulations were performed to extract the gamma‐ray spectrum. From each obtained spectrum's gamma‐ray peak, the photoelectric peak energy of Ep (57Co: 122 keV) and the peak width ΔEp at half the peak counting rate (full width at half maximum [FWHM]) were used in Equation (1) to calculate the energy resolution.

(1) Energyresolution=(ΔEp/Ep)×100(%).

### 2.3 Simulation evaluation

#### 2.3.1 Three‐dimensional brain phantom

For the construction of a digital phantom in the simulation of a three‐dimensional brain phantom, the actual phantom was scanned using CT (tube voltage: 120 kV, 80 mAs, matrix size: 512 × 512, pixel size: 1.27 mm, slice thickness: 3.0 mm). The header region was removed, and the data were compiled into a single file. This file was processed to fit the executable format of SIMIND, creating density and source maps for each region. To set the CT value of water to 1000 in the density maps, 1000 was added to each voxel. For the source maps, binarization processing was performed on each slice to extract each region and set the source areas. The source maps for each region were then overlaid, with CT values added and constructed as required.

Simulations for T‐SPECT and C‐SPECT were performed using SIMIND, with parameters validated through comparative verification using the small‐scale pixel detector. The simulation parameters for the detector and collimator used in this study are presented in Table 1. In particular, the simulation assumed the administration of 99mTc at 600 MBq and virtual SPECT acquisition using a low‐to‐medium energy high‐resolution (wide‐energy high‐resolution [WEHR]) collimator. The matrix size was set to 128 × 128, the magnification factor was 1.797 times, and the pixel size was 2.46 mm. The energy peak was set at 141 keV, the energy window was set at 15% (130.4–151.6 keV), and a low window of 3% was set for scatter correction (SC). The scattering window was set adjacent to the lower photopeak boundary.

**TABLE 1:** Simulation design specifications for detectors and collimators in C‐SPECT and T‐SPECT.

	Parameter		
Detector	Detector head radial position	Brain: 140 mm	Body: Variable
	Crystal material	**CZT**	**TlBr**
	Crystal size	510 × 390 mm	
	Detector array size I	208 pixels	
	Detector array size J	160 pixels	
	Crystal thickness	7.25 mm	5.00 mm
	Contact pad size	2.16 mm	
	Anode element pitch	2.46 mm	
	Intrinsic resolution (140 keV)	2.46 mm	
	Voltage	1000	500
	Mobility life (electrons)	3.0 × 10^−3^ cm^2^/V	30.0 × 10^−3^ cm^2^/V
	Mobility life (holes)	0.2 × 10^−3^ cm^2^/V	1.0 × 10^−3^ cm^2^/V
Collimator	Type	**WEHR**	**medium‐energy high‐resolution sensitivity (MEHRS)**
	Type of hole	Square	Hexagonal
	Hole length	45.00 mm	40.25 mm
	Hole diameter	2.26 mm	2.80 mm
	Septal thickness	0.2 mm	0.9 mm

Abbreviations: CZT, cadmium–zinc–telluride; TlBr, thallium bromide; SPECT, single‐photon emission computed tomography; WEHR, wide‐energy high‐resolution.

The rotation radius was 14 cm, 72 views were obtained at 5° steps, and the total acquisition times were set to 5.0, 7.0, 10.0, 20.0, and 30.0 min. Furthermore, a reference image with substantially reduced statistical noise was obtained at 120.0 min (Figure 2).

The acquired data were reconstructed using the filtered back projection (FBP) method and ordered subset expectation maximization (OSEM) method (6 subsets, 10 iterations).^27^ The corrections included attenuation correction (AC) and SC. For smoothing, a pre‐Butterworth filter (cutoff: 0.50 cycles/cm, order: 8) was used. AC was performed using the Chang method, and SC was performed using the dual‐energy window (DEW) method.

The energy resolution was calculated from the energy spectra of the 30 min acquisition reconstructed data for each device. Next, a count profile in the horizontal direction at the center of the transverse slice level with the most extracted basal angle for each acquisition time was calculated. Using the 120 min acquisition images in which the statistical noise was sufficiently reduced as reference images, the peak signal‐to‐noise ratio (PSNR) and structural similarity (SSIM)^28^ were calculated for each acquisition time image. The Friedman test was performed for statistical analysis, and the Dunn test was used for post‐hoc testing. Significant differences in the normalized calculated values between T‐SPECT and C‐SPECT for different acquisition times were determined, and the significance level was set at *p* < 0.001. PSNR and SSIM were calculated using the following equations.

(2) $$\mathrm{PSNR}=10\cdot \mathrm{lo}g_{10}\left(\frac{\mathrm{ma}x^{2}}{\mathrm{MSE}}\right)\;\left[\mathrm{db}\right]$$

here, max represents the maximum value of the reference image, and MSE (mean squared error) represents the squared difference between each pixel in reference and comparison images, averaged over all pixels.

(3) $$\mathrm{SSIM}\left(x,y\right)=\frac{\left(2\mu_{x}\mu_{y}+C_{1}\right)\left(2\sigma_{xy}+C_{2}\right)}{\left(\mu_{x}^{2}+\mu_{y}^{2}+C_{1}\right)\left(\sigma_{x}^{2}+\sigma_{y}^{2}+C_{2}\right)}$$

here, 𝑥 is the reference image, *y* is the comparison image, $\mu_{x},\mu_{y}$ are local mean values of each image, $\sigma_{x},\sigma_{y}$ are local standard deviations for each image, $\sigma_{xy}$ is covariance, $C_{1},C_{2}$ are constant, $C_{1}={\left(K_{1}L\right)}^{2},C_{2}={\left(K_{2}L\right)}^{2},L$ is $255\;\left(\mathrm{dynamic}\;\mathrm{range}\;\mathrm{of}\;\mathrm{an}\;8-\mathrm{bit}\;\mathrm{image}\right),\;K_{1}$ is 0.01, and $K_{2}$ is 0.03.

Furthermore, for frequency domain evaluation, each acquired image was subjected to a two‐dimensional Fourier transform, and the power spectrum density (PSD) was calculated to evaluate the two‐dimensional frequency distribution one dimensionally.^29^

### 2.4 NEMA‐IEC body phantom

The phantom used in the simulation was designed to accommodate hot spheres with diameters of 37, 28, 22, 17, 13, and 10 mm, and the geometric arrangement was set according to the NEMA standards (Figure 2). A digital phantom was obtained under the same processing conditions as a three‐dimensional brain phantom. Considering that the 208‐keV gamma ray emitted from 177Lu has a high emission ratio (11.0%), the energy peak was set to 208 keV. The processing conditions included a matrix size of 256 × 256, a magnification factor of 0.898 times, and a pixel size of 2.46 mm, with an energy window of 15% (192.4–223.6 keV) and a low window of 3% for SC. The scattering window was set adjacent to the lower photopeak boundary. The acquisition was performed with automatic proximity, 120 views at 3° steps, and total acquisition times of 3.0, 5.0, 10.0, 15.0, 20.0, and 30.0 min using a medium‐energy, high‐resolution sensitivity collimator.^10^ T‐SPECT and C‐SPECT acquisitions were obtained under these conditions. In this study, a tumor within healthy liver tissue was simulated by assuming a radioactivity concentration 72 h after the administration of 7.4 GBq of 177Lu (tumor/liver: 0.972/0.117 MBq/mL), setting the hot sphere‐to‐background (BG) ratio to 8:1.^30^

The acquired data were reconstructed using the resolution‐corrected OSEM method (10 subsets, 4 iterations)^31^, ^32^ on a Xeleris workstation. The corrections included AC and SC. For smoothing, a post‐Butterworth filter (cutoff: 0.50 cycles/cm, power: 16) was used. AC was performed using the CT‐AC method, and SC was performed using the DEW method.

For evaluation, energy resolution was calculated from the energy spectra of the 30 min acquisition reconstructed data for each device. Next, the contrast recovery coefficient (CRC) and contrast‐to‐noise ratios (CNRs) were quantitatively evaluated on the transaxial images of three slices above and below the central slice level of the hot spheres in the reconstructed data. CRC and CNR were calculated using the following equations:

(4) $$\text{CR}C_{H,j}\frac{C_{H,j}/C_{B}^{-1}}{A_{H}/A_{B}^{-1}}\times 100\left[\%\right]$$

here, *C_H,j_* represents the average count of the hot sphere of size *j*, *C_B_* is the average count of 12 BG regions of interest (ROIs), *A*_𝐻_ is the true radioactivity concentration of the hot sphere, and *A*_B_ is the true radioactivity concentration of the BG.

(5) $$\mathrm{CNR}\;=\frac{C_{H,j}-C_{B}}{SD_{B,\mathrm{mean}}}$$

here, SD*_B_*_,mean_ represents the mean standard deviation of the counts of 12 BG ROIs.

Furthermore, PSD was calculated for each acquired image.

## 3 RESULTS

### 3.1 Small‐scale pixel detector

**FIGURE 3:**
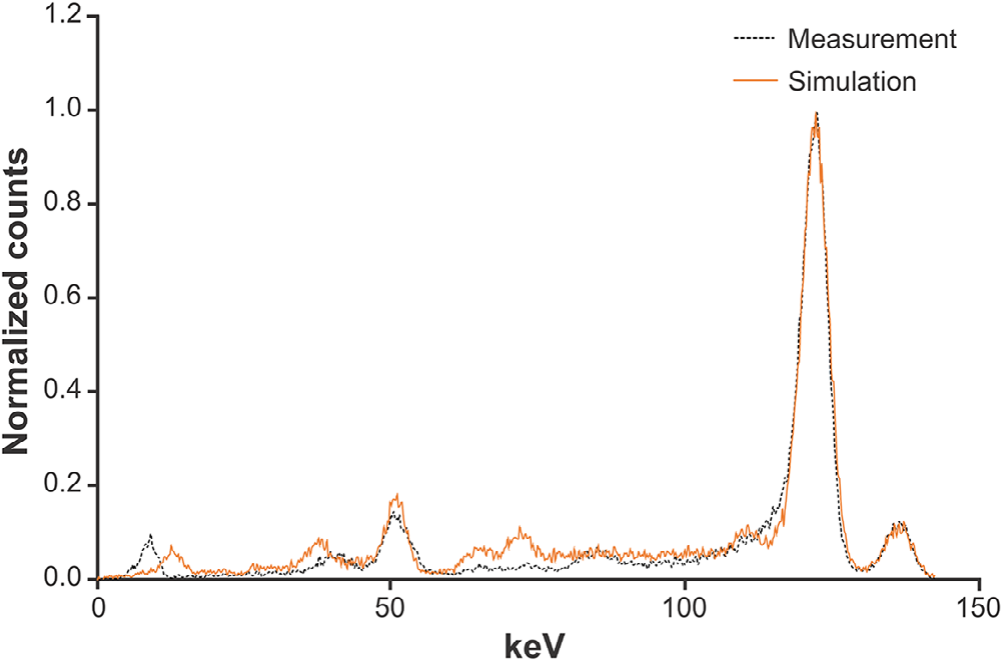
Energy peak of actual measurement and simulation of 57Co in a small‐scale TlBr pixel detector. TlBr, thallium bromide.

Figure 3 shows the energy spectra of 57Co obtained for actual acquisition and simulation using a small‐scale pixel detector. The simulation peak approximated the actual measurement, including the Tl escape peak on the low‐energy side. For the actual measurement, the FWHM was 5.38 keV, resulting in an energy resolution of 4.41%. Similarly, for the simulation, the FWHM was 5.30 keV, resulting in an energy resolution of 4.34%.

### 3.2 Three‐dimensional brain phantom

**FIGURE 4:**
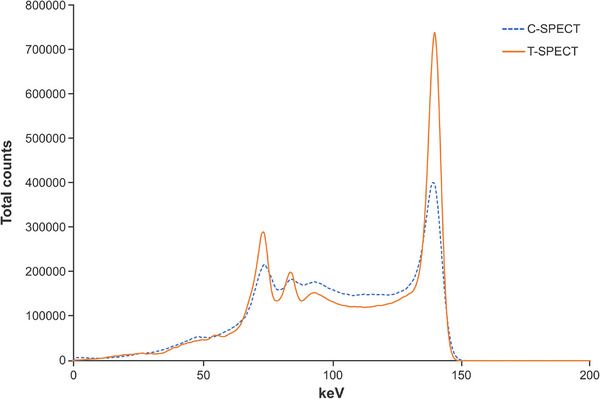
Energy peak of 99mTc three‐dimensional brain phantom in each method. The sensitivity of the system for the 99mTc three‐dimensional brain phantom was 43.0 and 32.6 Cps/MBq for T‐SPECT and C‐SPECT. SPECT, single‐photon emission computed tomography.

Figure 4 shows the energy spectra at a 30 min acquisition time for each device. In T‐SPECT, the relative counts in the Compton region, determined from the photopeak counts, were lower than those in C‐SPECT, and the relative counts of the escape peak in the low‐energy region were lower than those in the corresponding region of C‐SPECT. From the energy spectra in Figure 4, the FWHM for T‐SPECT was 6.35 keV, resulting in an energy resolution of 4.50%. In contrast, for C‐SPECT, the FWHM was 10.35 keV, resulting in an energy resolution of 7.34%.

The sensitivity of the system for the 99mTc three‐dimensional brain phantom was 43.0 and 32.6 Cps/MBq for T‐SPECT and C‐SPECT.

**FIGURE 5:**
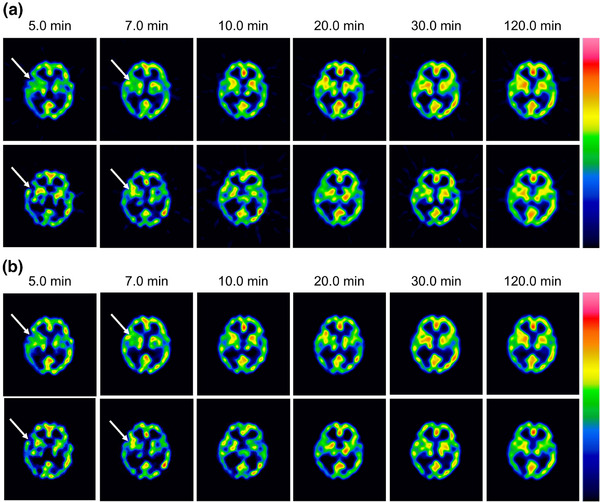
Reconstructed images at each total acquisition time for 99mTc three‐dimensional brain phantom. (a) FBP reconstruction images (upper: T‐SPECT, lower: C‐SPECT). (b) OSEM reconstruction images (upper: T‐SPECT, lower: C‐SPECT). FBP, filtered back projection; OSEM, ordered subset expectation maximization; SPECT, single‐photon emission computed tomography. In each reconstruction method, even with a short acquisition time, T‐SPECT maintains sufficient counts in the basal ganglia region, thereby resulting in better contrast resolution.

**FIGURE 6:**
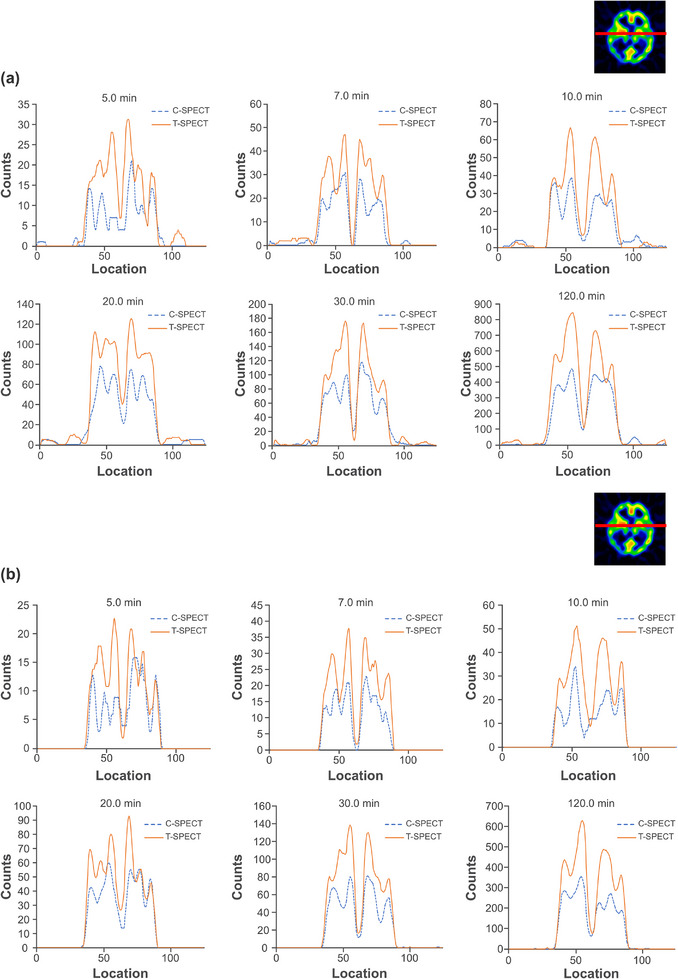
Count profile of the basal ganglia at each acquisition time. (a) FBP reconstruction. (b) OSEM reconstruction. (Top right corner: slice position of the count profile). FBP, filtered back projection; OSEM, ordered subset expectation maximization.

**FIGURE 7:**
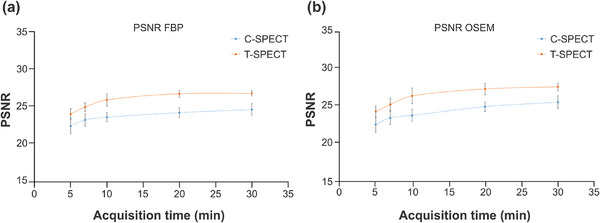
PSNR of each total count in each process. (a) FBP reconstruction. (b) OSEM reconstruction. FBP, filtered back projection; OSEM, ordered subset expectation maximization; PSNR, peak signal‐to‐noise ratio.

**FIGURE 8:**
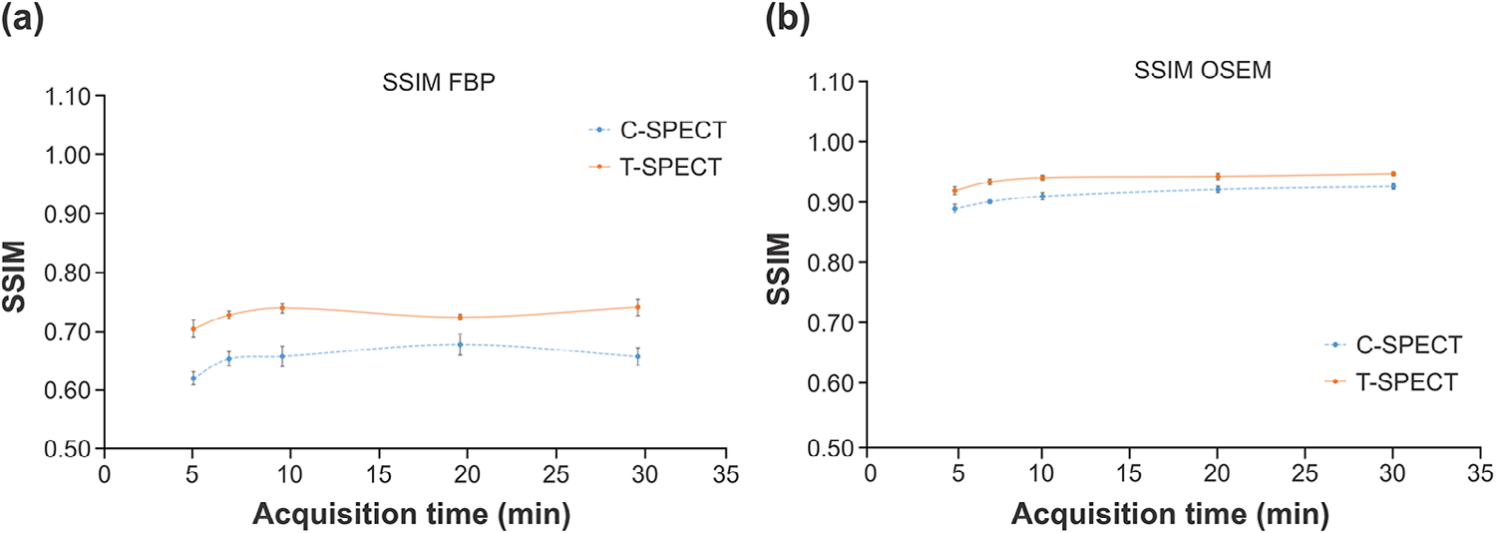
SSIM of each total count in each process. (a) FBP reconstruction. (b) OSEM reconstruction. FBP, filtered back projection; OSEM, ordered subset expectation maximization; SSIM, structural similarity.

Figure 5 shows reconstructed images of the individual acquisition times for each device, and Figure 6 shows the count profiles of the basal ganglia. T‐SPECT demonstrated count densities that were 1.42–1.52 times higher than C‐SPECT in the highest accumulation area of the basal ganglia at each acquisition time. The PSNR transition is shown in Figure 7, and the results are presented in Table 2. The PSNR was statistically significantly higher for acquisition times ranging from 5 to 20 min. The SSIM transition is shown in Figure 8, and the results are presented in Table 3. The SSIM was statistically significantly higher for acquisition times ranging from 5 to 20 min.

**TABLE 2:** Statistical analysis of PSNR for each method using (a) FBP and (b) OSEM reconstruction.

(a)						
		5.0 (min)	7.0 (min)	10.0 (min)	20.0 (min)	30.0 (min)
PSNR	T‐SPECT	24.15	25.07	26.05	26.87	26.91
	C‐SPECT	22.53	23.34	23.71	24.32	24.75
*p*‐value	vs. 120.0 (min)	< 0.0001	< 0.0001	< 0.0001	< 0.0001	< 0.0001
(b)						
		5.0 (min)	7.0 (min)	10.0 (min)	20.0 (min)	30.0 (min)
PSNR	T‐SPECT	24.35	25.28	26.43	27.35	27.64
	C‐SPECT	22.61	23.50	23.83	24.98	25.59
*p*‐value	vs. 120.0 (min)	< 0.0001	< 0.0001	< 0.0001	< 0.0001	< 0.0001

Abbreviations: FBP, filtered back projection PSNR, peak signal‐to‐noise ratio; OSEM, ordered subset expectation maximization; SPECT, single‐photon emission computed tomography.

**TABLE 3:** Statistical analysis of SSIM for each method using (a) FBP and (b) OSEM reconstruction.

(a)						
		5.0 (min)	7.0 (min)	10.0 (min)	20.0 (min)	30.0 (min)
SSIM	T‐SPECT	0.70	0.73	0.74	0.72	0.74
	C‐SPECT	0.62	0.65	0.66	0.68	0.66
*p*‐value	vs. 120.0 (min)	< 0.0001	< 0.0001	< 0.0001	< 0.0001	< 0.0001
(b)						
		5.0 (min)	7.0 (min)	10.0 (min)	20.0 (min)	30.0 (min)
SSIM	T‐SPECT	0.93	0.94	0.95	0.95	0.95
	C‐SPECT	0.90	0.91	0.92	0.93	0.93
*p*‐value	vs. 120.0 (min)	< 0.0001	< 0.0001	< 0.0001	< 0.0001	< 0.0001

Abbreviations: FBP, filtered back projection; OSEM, ordered subset expectation maximization; SPECT, single‐photon emission computed tomography; SSIM, structural similarity.

**FIGURE 9:**
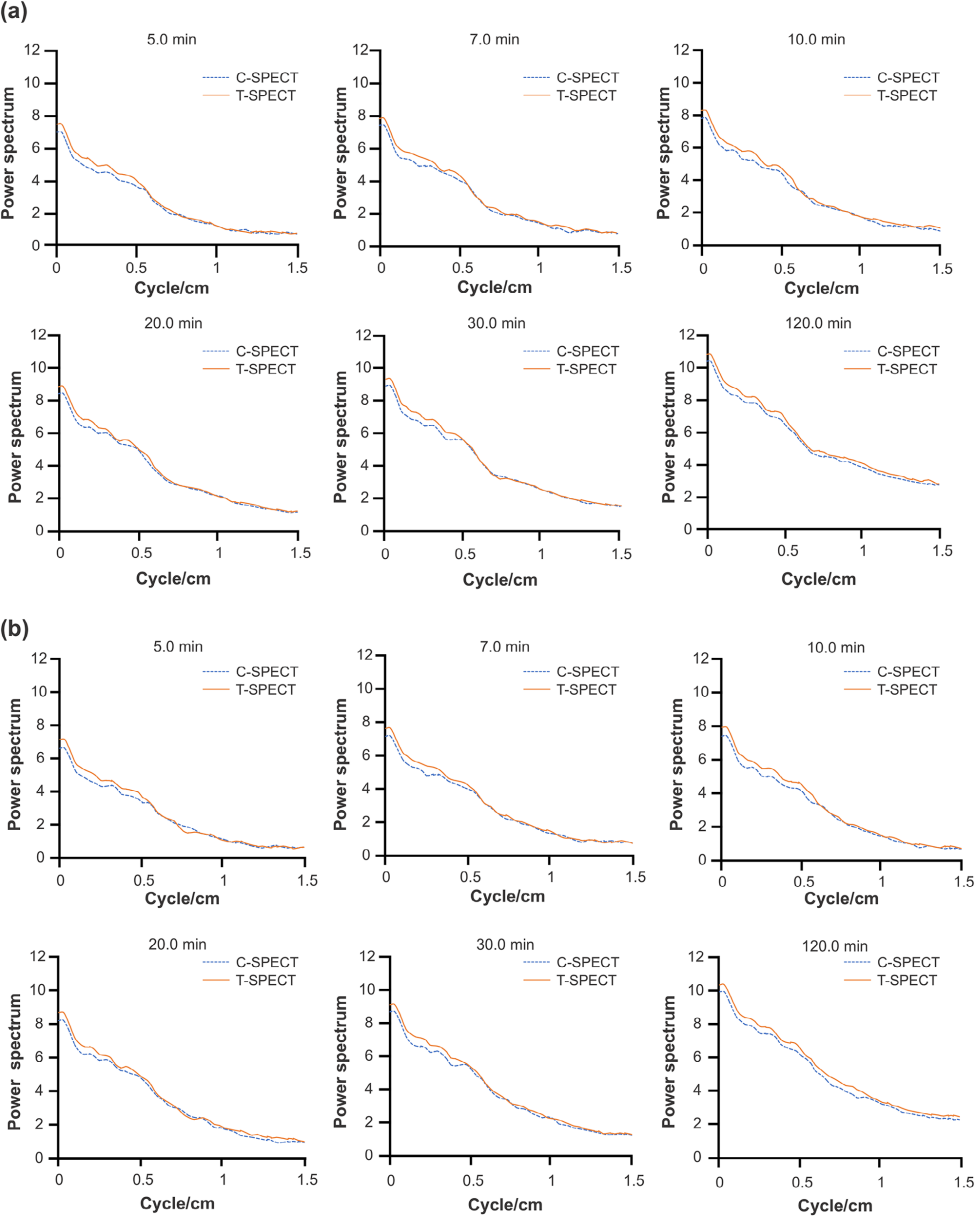
Power spectrum density of 99mTc three‐dimensional brain phantom images by each method.

Finally, PSD is shown in Figure 9. At each acquisition time, T‐SPECT demonstrated higher PSD values in the low‐frequency region than C‐SPECT, whereas both were equivalent in the high‐frequency region.

### 3.3 NEMA‐IEC body phantom

**FIGURE 10:**
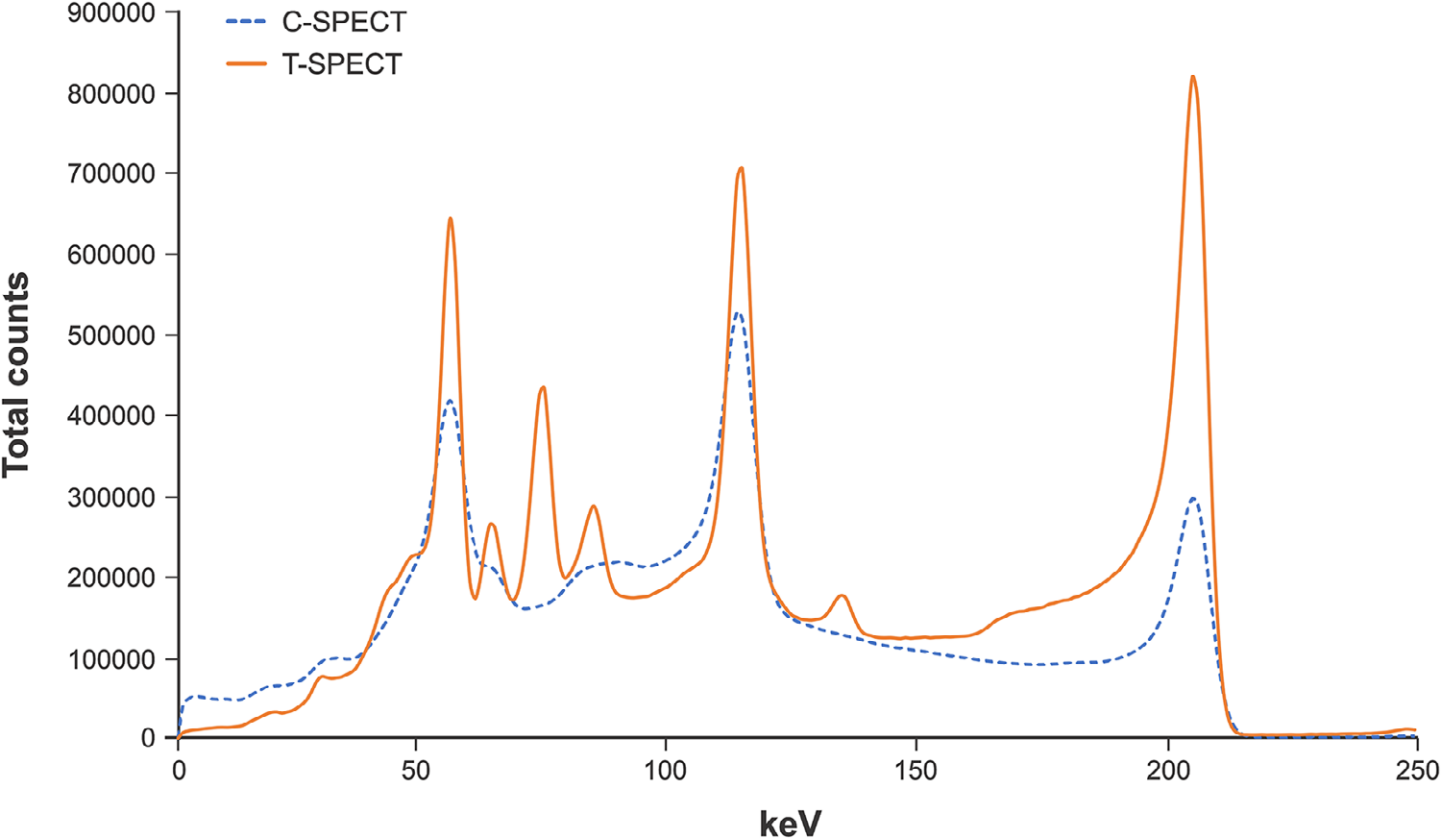
Energy peak of 177 Lu NEMA‐IEC body phantom in each method. NEMA, National Electrical Manufacturers Association.

**FIGURE 11:**
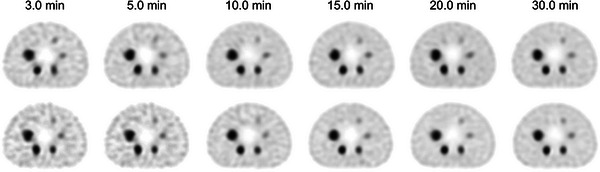
Images at each acquisition time for 177 Lu NEMA‐IEC body phantom. (Upper: T‐SPECT, lower: C‐SPECT). NEMA, National Electrical Manufacturers Association; SPECT, single‐photon emission computed tomography.

**FIGURE 12:**
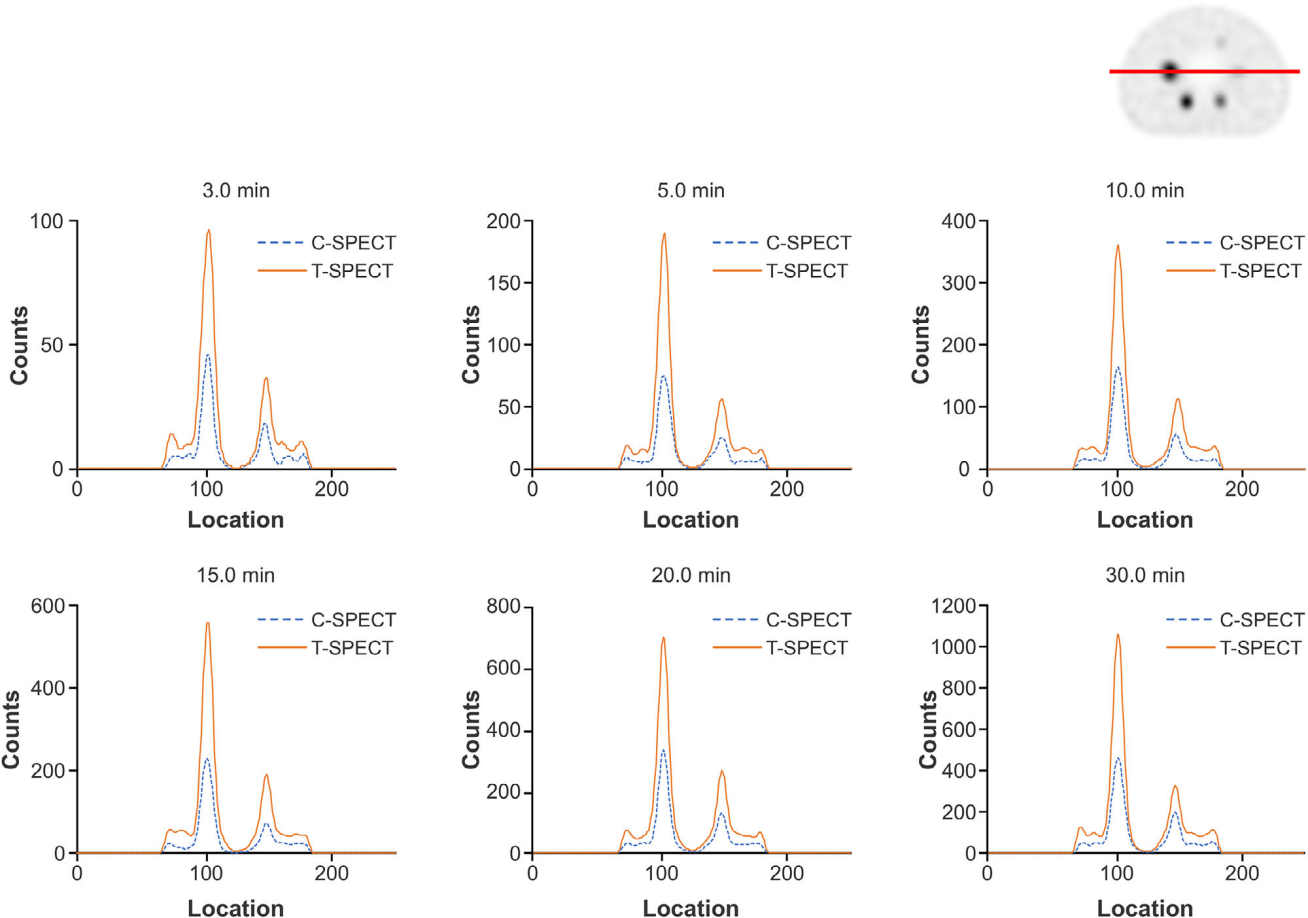
Count profiles of spheres with sizes of 17 and 37 mm at each collection time in 177Lu. (Top right corner: slice position of the count profile).

**FIGURE 13:**
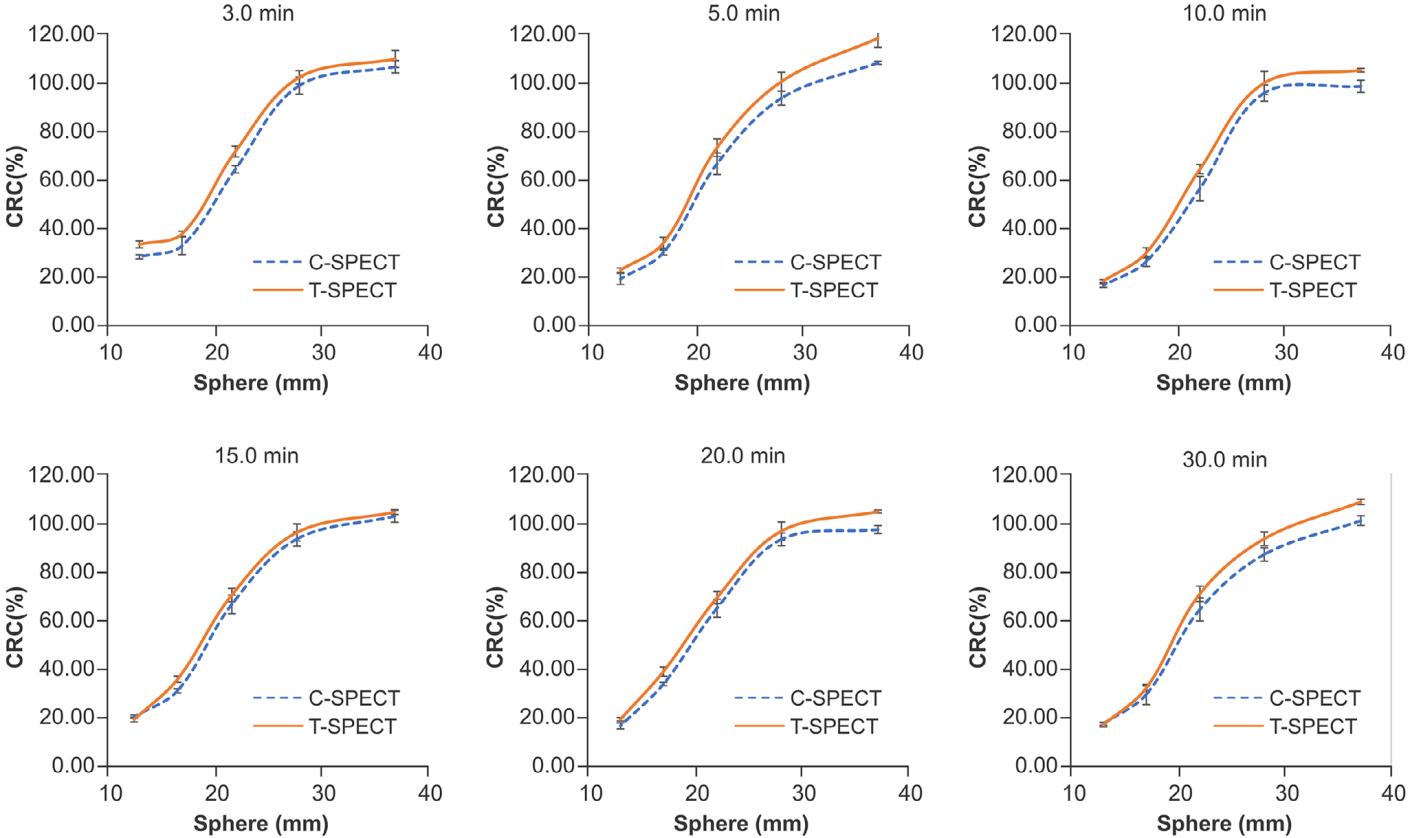
CRC at each acquisition time for each method. CRC, contrast recovery coefficient.

**FIGURE 14:**
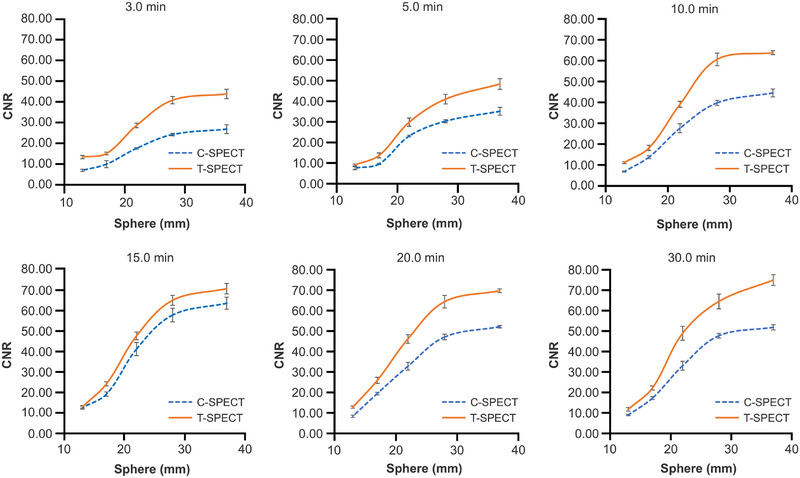
CNR at each acquisition time for each method. CNR, contrast‐to‐noise ratio.

Figure 10 shows the energy spectra at a 30‐min acquisition time for each device. Compared with C‐SPECT, T‐SPECT demonstrated a reduction in relative counts in the Compton region based on the 208‐keV photopeak counts and exhibited a similar trend at the 113‐keV peak. At 208 keV, the FWHM for T‐SPECT was 8.19 keV, resulting in an energy resolution of 3.94%. In contrast, for C‐SPECT, the FWHM was 10.82 keV, resulting in an energy resolution of 5.20%. Figure 11 shows the reconstructed images for each acquisition time, and Figure 12 shows the count profiles for 17‐mm^33^, ^34^ spheres and 37‐mm spheres. Compared with C‐SPECT, T‐SPECT demonstrated 1.90–3.06 times higher counts in the highest accumulation area of the 37‐mm sphere at each acquisition time. Figures 13 and 14 present the CRC and CNR, respectively, for T‐SPECT and C‐SPECT. T‐SPECT showed higher values than C‐SPECT at each acquisition time.

The sensitivity of the system for the 177Lu NEMA‐IEC body phantom was 7.53 and 3.71 Cps/MBq for T‐SPECT and C‐SPECT.

**FIGURE 15:**
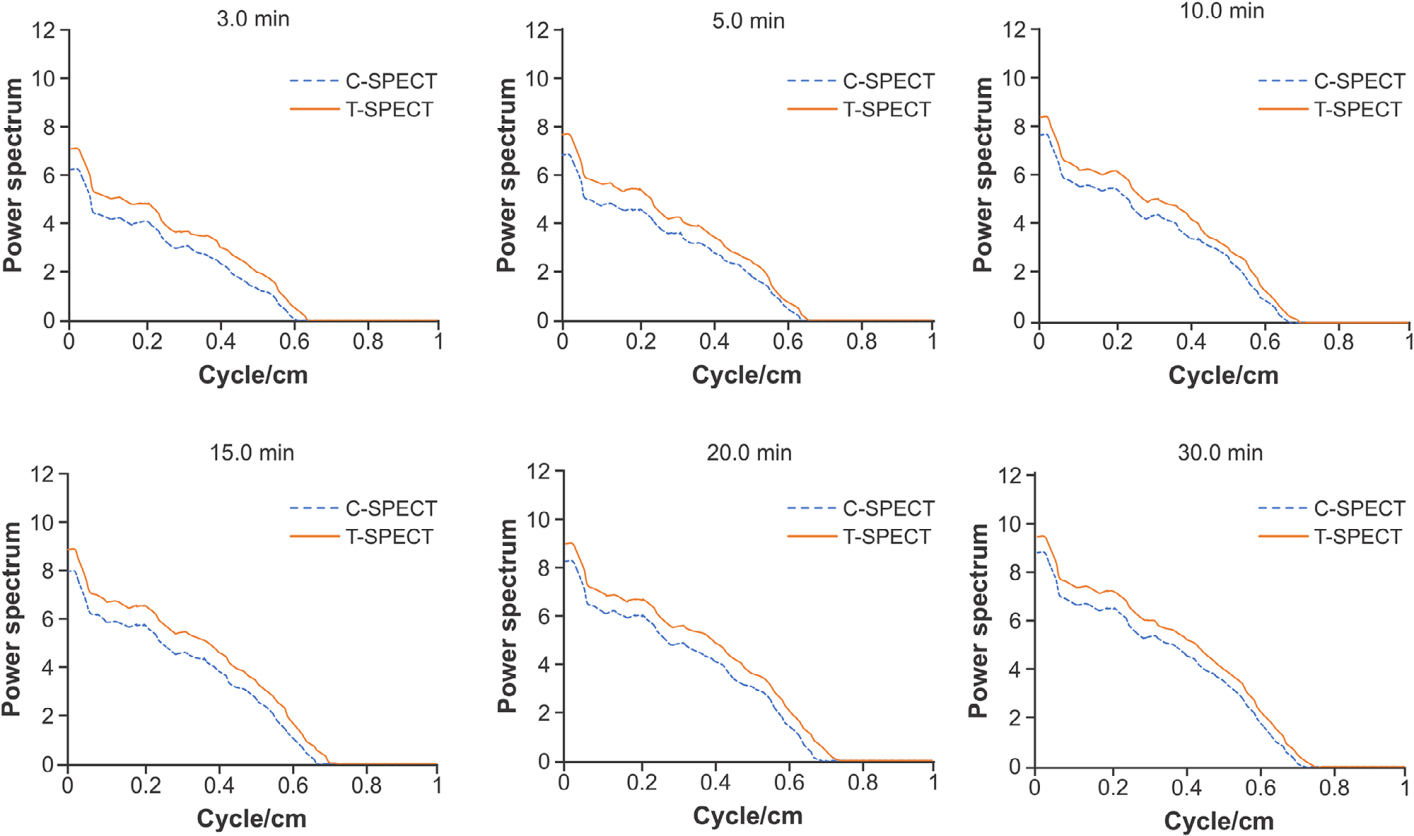
Power spectrum density of 177Lu NEMA‐IEC body phantom images by each method. NEMA, National Electrical Manufacturers Association.

Next, PSD is shown in Figure 15. At each acquisition time, T‐SPECT demonstrated higher values in the low‐frequency region than C‐SPECT, whereas T‐SPECT and C‐SPECT were equivalent in the high‐frequency region.

## 4 DISCUSSION

This study used Monte Carlo simulation to evaluate the clinical usefulness of T‐SPECT, a whole‐body SPECT system equipped with TlBr that is expected to have high detection efficiency because of its high atomic number and density in comparison with C‐SPECT. Furthermore, the characteristics of imaging with different radionuclides were evaluated using 99mTc and 177Lu.

First, as shown in Figure 3, the actual acquisition and simulation using the SIMIND Monte Carlo program for 57Co with a 2 × 2 pixel detector of 1 × 1 mm^2^ demonstrated almost identical energy spectra and the energy resolution in actual acquisition and simulation. The accuracy of reproducing the semiconductor compound‐specific hole tailing^5^, ^35^, ^36^ observed in the actual acquisition can be attributed to this result. The Tl escape peak^17^, ^37^ of approximately 50 keV was also accurately reproduced, suggesting that the carrier transport was well modeled.^38^ Therefore, the simulation in this study closely approximated the actual measurements, providing a highly accurate guide for future T‐SPECT development. However, discrepancies were observed between the simulation and actual measurements in the low‐energy range. These differences are potentially because of variations in the modeling of the characteristic x‐rays of the collimator and the scattered radiation from external sources. Using these optimized simulation parameters, the clinical applicability of brain perfusion imaging using 99mTc, which is a radionuclide commonly used in SPECT imaging, was considered from qualitative images simulating clinical conditions.

The energy spectra of C‐SPECT and T‐SPECT shown in Figure 4 confirm that T‐SPECT significantly reduced the impact of hole tailing compared with C‐SPECT. Furthermore, the energy spectra of T‐SPECT improved because of the high detection efficiency characteristic of TlBr. The photopeak count of T‐SPECT was 1.71 times higher, indicating improved sensitivity compared with that using C‐SPECT. The peaks were observed around 73 and 83 keV, corresponding to the Kα and Kβ x‐rays of Tl. The larger peak at around 73 keV is attributed to the greater occurrence of Kα x‐rays. Moreover, the escape peak around 67 keV, which results from the subtraction of the Kα x‐ray energy from the photopeak due to the escape of Kα x‐rays from the detector, is effectively absorbed by the 73‐keV Kα peak. As shown in Figure 5, T‐SPECT achieved higher total uptake because of its increased detection efficiency, ensuring sufficient counts even with shorter acquisition times than those used in the reference images. The count profiles of the basal ganglia in Figure 6 show that T‐SPECT improved contrast resolution in the deep regions, probably because of the superior gamma‐ray absorption efficiency. This was additionally true for the FBP reconstruction method, reported as optimal for cerebral blood flow assessment in C‐SPECT,^27^ as well as for the OSEM reconstruction method designed to improve clinical quantification in Anger‐type SPECT systems.^39^, ^40^

Figures 7 and 8 show that T‐SPECT demonstrated higher PSNR and SSIM values across all acquisition times than C‐SPECT, with statistically significant differences. This suggests that T‐SPECT combines high sensitivity from superior gamma‐ray absorption efficiency with high contrast resolution from reduced hole tailing and improved energy resolution, even with short acquisition times. Furthermore, this study used SSIM as an evaluation index, which is strongly correlated with visual assessment. The significant difference observed in SSIM compared with C‐SPECT suggests its potential usefulness in qualitative visual assessment during image interpretation.

From the results in Figure 9, T‐SPECT demonstrated higher values in the low‐frequency region than C‐SPECT, indicating a more accurate reproduction of internal image information. In the high‐frequency region, T‐SPECT was almost equivalent to C‐SPECT. This result was attributed to the smoothing process with a cutoff of 0.50 cycles/cm, which effectively removed high‐frequency components beyond the fundamental frequency, resulting in similar contour formation reproducibility for T‐SPECT and C‐SPECT.

Next, we considered the applicability of quantitative evaluation in 177Lu imaging. 177Lu‐labeled radiopharmaceuticals, such as 177Lu‐DOTATATE and 177Lu‐prostate‐specific membrane antigen used in nuclear medicine therapy with beta‐rays for neuroendocrine tumors and castration‐resistant prostate cancer, respectively, are clinically used. These pharmaceuticals emit 208‐keV (11.0%) and 113‐keV (6.4%) gamma rays, making imaging possible, and are attracting attention as theranostic radiopharmaceuticals.^41^, ^42^, ^43^, ^44^

As shown in Figure 10, T‐SPECT achieved a photopeak count of 208 keV, which was 2.76 times higher than that of C‐SPECT. This improvement compared with that observed for 99mTc (at 1.71 times) can be attributed to the differences in photoelectric absorption and hole tailing between the detectors, including the detector thickness used in this study. Therefore, 177Lu imaging with T‐SPECT is expected to have significantly enhanced sensitivity compared with 177Lu imaging with C‐SPECT. Furthermore, similar to 99mTc, T‐SPECT significantly reduced the impact of hole tailing at 208 keV compared with C‐SPECT, indicating improved energy resolution.

Furthermore, T‐SPECT exhibited an escape peak near 135 keV, corresponding to the Tl Kα escape peak from the 208‐keV gamma ray. The Kβ escape peak at 125 keV is absorbed and extracted on the high‐energy side of the 113‐keV peak. Additionally, when the 113‐keV gamma ray interacts with Tl, the Tl‐Kα and Tl‐Kβ escape peaks are slightly observed around 40 and 30 keV, respectively. Next, the peaks at 64, 73, and 83 keV are considered. Not only does the target pixel exhibit an escape peak from Tl at 113 keV, but gamma rays also interact with Tl atoms in surrounding pixels to generate characteristic x‐rays.^45^ Considering the Tl characteristic x‐rays Kα at 73 keV and Kβ at 83 keV, the observed peaks are consistent. The larger 73‐keV peak was attributed to the higher occurrence of Kα. Thus, the influence of the characteristic x‐rays added to the escape peak can be observed at 73 and 83 keV. Moreover, the peaks observed around 56 and 64 keV are attributed to the characteristic x‐rays of Hf‐Kα and Hf‐Kβ from 177Hf. The 64‐keV peak may also be influenced by characteristic x‐rays originating from the collimator. This observation is consistent with the slight peak observed on the higher‐energy side of the 56‐keV peak in C‐SPECT.

Next, Figure 11 visually confirms that T‐SPECT maintains sufficient counts even with shorter acquisition times than C‐SPECT. Furthermore, Figure 12 shows that the peak counts of T‐SPECT are approximately twice those of C‐SPECT, indicating improved detection capability for small accumulations.

Next, as shown in Figures 13 and 14, T‐SPECT demonstrated higher CRC and CNR values for all spheres than C‐SPECT, which was consistent across all acquisition times. In cases of highly malignant neuroendocrine tumors, CNR increases as the tumor size grows.^46^, ^47^ T‐SPECT and C‐SPECT demonstrated similar trends. However, T‐SPECT exhibited higher values compared with C‐SPECT, indicating that T‐SPECT achieves high sensitivity and contrast resolution quantitatively and suggests the possibility of shorter acquisition times. The reasons for the results in Figure 15 are similar to those for Figure 9.

This study has several limitations. First, the TlBr detector size was fixed at 2.46 mm with a thickness of 5.0 mm. Lee and Park demonstrated through simulations that adjusting detector thickness can improve sensitivity and spatial resolution.^48^ Further investigations into the relationship between the optimal pixel size and detector thickness can lead to more accurate charge collection efficiency.^49^ Moreover, detector thickness may influence the scatter fraction. While we previously evaluated the scatter fraction of C‐SPECT and Velo et al.^50^ assessed the primary‐to‐scattered ratio in C‐SPECT systems, we have not evaluated this for T‐SPECT detectors because of the ongoing system development. Further image quality improvements may be achievable through scatter fraction assessment and appropriate correction methods. Second, the enhanced energy resolution compared with C‐SPECT suggests that further narrowing the acquisition energy window could result in higher contrast resolution. Furthermore, the simulation parameters used in this study were based on actual measurements with a 57Co source. Future work involving the parameter settings for each radionuclide is expected to further enhance simulation accuracy. Additionally, compared to C‐SPECT, T‐SPECT can narrow the energy acquisition window due to reduced hole tailing; this adjustment can improve contrast resolution while maintaining sensitivity, suggesting the possibility of better imaging quality.

The SIMIND Monte Carlo program used in this study accurately reproduced carrier transport.^38^ Therefore, data that are very similar to actual measurements can be obtained, providing an important guide for constructing appropriate detectors for the development of T‐SPECT systems. In our previous report on C‐SPECT, we detailed the configuration of SC to address the effects of hole tailing on scatter radiation, as validated by actual measurements.^51^ Given that T‐SPECT is less affected by hole tailing compared to C‐SPECT, it is anticipated that SC settings for T‐SPECT may differ. Therefore, the SIMIND Monte Carlo program, which can be accurately tuned to actual measurements and simulate ideal conditions, is also valuable for verifying data acquisition and correction processes. Future work will verify each reconstruction and acquisition method in detail, assessing clinical applicability by incorporating clinical metrics to guide optimal device design.

## 5 CONCLUSIONS

In this study, the clinical applicability of T‐SPECT was evaluated by comparing it with C‐SPECT using simulation techniques with parameter settings that accurately reproduced actual measurements, including carrier transport. In imaging with 99mTc and 177Lu radionuclides, T‐SPECT demonstrated improved contrast resolution because of its superior energy resolution and high sensitivity because of its larger linear attenuation coefficient than C‐SPECT. Furthermore, using these simulation techniques, the evaluation of detector structures, detector arrays, and collimator compatibility in the development of T‐SPECT is possible.

## CONFLICT OF INTEREST STATEMENT

The authors declare no conflicts of interest.
